# Changes in functional connectivity after theta-burst transcranial magnetic stimulation for post-traumatic stress disorder: a machine-learning study

**DOI:** 10.1007/s00406-020-01172-5

**Published:** 2020-07-27

**Authors:** Amin Zandvakili, Hannah R. Swearingen, Noah S. Philip

**Affiliations:** 1 Department of Psychiatry and Human Behavior, Alpert Medical School of Brown University, Providence, RI 02906 USA; 2 VA RR&D Center for Neurorestoration and Neurotechnology, Providence VA Medical Center, 830 Chalkstone Ave, Providence, RI 02908 USA

**Keywords:** EEG, Theta burst, Delta, Transcranial magnetic stimulation, Post-traumatic stress disorder

## Abstract

Intermittent theta burst stimulation (iTBS) is a novel treatment approach for post-traumatic stress disorder (PTSD), and recent neuroimaging work indicates that functional connectivity profiles may be able to identify those most likely to respond. However, prior work has relied on functional magnetic resonance imaging, which is expensive and difficult to scale. Alternatively, electroencephalography (EEG) represents a different approach that may be easier to implement in clinical practice. To this end, we acquired an 8-channel resting-state EEG signal on participants before (*n* = 47) and after (*n* = 43) randomized controlled trial of iTBS for PTSD (ten sessions, delivered at 80% of motor threshold, 1,800 pulses, to the right dorsolateral prefrontal cortex). We used a cross-validated support vector machine (SVM) to track changes in EEG functional connectivity after verum iTBS stimulation. We found that an SVM classifier was able to successfully separate patients who received active treatment vs. sham treatment, with statistically significant findings in the Delta band (1–4 Hz, *p* = 0.002). Using Delta coherence, the classifier was 75.0% accurate in detecting sham vs. active iTBS, and observed changes represented an increase in functional connectivity between midline central/occipital and a decrease between frontal and central regions. The primary limitations of this work are the sparse electrode system and a modest sample size. Our findings raise the possibility that EEG and machine learning may be combined to provide a window into mechanisms of action of TMS, with the potential that these approaches can inform the development of individualized treatment methods.

**Electronic supplementary material:**

The online version of this article (10.1007/s00406-020-01172-5) contains supplementary material, which is available to authorized users.

## Introduction

Posttraumatic stress disorder (PTSD) is a highly prevalent psychiatric condition marked by trauma exposure. It is often associated with severe impairment of occupational and social functioning and diminished quality of life [7,12,24]. There is an increased need for the development of novel treatments for PTSD, specifically for the Veteran population, as current PTSD treatments, such as psychotherapy and pharmacotherapy, may not be as effective in symptom reduction in Veterans compared to the general population [25]. Neuromodulation interventions, such as repetitive transcranial magnetic stimulation (rTMS, hereafter TMS), are proving to be an effective treatment for pharmacoresistant major depressive disorder (MDD) [4,16] and PTSD [3,13]. TMS provides a pulsed magnetic field, typically for the left dorsolateral prefrontal cortex (DLPFC) for MDD, which induces an electric current to modulate activity in that brain region. Though effective, standard TMS treatment can be cumbersome and time-consuming. Currently available protocols generally require at least 30 min, delivered every day, for up to six weeks. New treatment protocols for TMS delivery are being introduced with shorter treatment durations and comparable effectivity. One such stimulation protocol, Theta-burst stimulation (TBS), is effective in both depressed patients [1] and those with PTSD [20].

In TBS, bursts of high-frequency (50 Hz) stimulation repeated at 5 Hz (200 ms intervals) is used. These pulses are thought to modulate synaptic plasticity [10], and stimulation can be delivered in an intermittent (iTBS) or continuous fashion, where the two approaches are thought to have excitatory and inhibitory effects, respectively. TBS can be hypothesized to be particularly effective for the treatment of PTSD due to the stimulation pattern resembling the theta oscillations of hippocampal memory systems, thus relating to PTSD’s intrusive traumatic memories [14]. Despite these, the exact mechanism of TBS treatment is not fully understood. Prior imaging studies of TBS have indicated that pretreatment functional connectivity, utilizing functional magnetic resonance imaging, can predict clinical response to stimulation in depression (reviewed in [19] and PTSD [21]. However, because fMRI is associated with high costs that limit general clinical use, several alternatives to MRI are being developed, foremost among them electroencephalography (EEG), which may provide a more scalable technology to use in clinical contexts. EEG has now been used to identify potential signatures related to PTSD [5], in a prior study, we were able to identify EEG predictors of symptom response to TMS using data from a sparse electrode system [29]. Here, we attempted to use EEG to track changes in brain functional connectivity associated with iTBS treatment in patients with PTSD.

To this end, we recorded resting-state eyes-closed EEG before and after a course of sham-controlled iTBS PTSD [20]. We used this data to train a machine-learning algorithm to track changes in EEG connectivity associated with TBS treatment, hypothesizing this algorithm could reliably identify neural signals associated with verum stimulation, compared to sham.

## Methods

We used the EEG data recorded during a previously published randomized, double-blind study of iTBS for PTSD, conducted at the Providence VA Medical Center in Providence, RI, USA. For full details of the study, including inclusion/exclusion criteria, clinical outcomes, and initial fMRI results, see [17,20]. In brief, all participants met DSM-5 criteria for PTSD and eligibility to receive TMS treatment, and any medications and psychotherapeutic treatments were stable for at least 6 weeks before beginning study procedures. After randomization and motor threshold determination, sham-controlled iTBS was delivered to the right dorsolateral prefrontal cortex (DLPFC) daily for ten business days via a Magstim Rapid 2 + 1 system (Magstim, Whitland, U.K.). Separate (and double-blinded) stimulation coils were used for sham and active stimulation. The sham coil was designed to mimic the sound and sensory feelings associated with transcranial magnetic stimulation but with weaker e-field. Stimulation was delivered at 80% of active motor threshold, for 1800 pulses and 9.5 min. In that study, there was a statistically significant and clinically meaningful improvement in social and occupational function at two weeks, with clinically meaningful effect sizes at the end of the two weeks. EEG was obtained after randomization, and then after the final iTBS session. Of the 50 participants in the intent-to-treat (ITT) sample, pretreatment EEG was obtained from 47 participants, and post-treatment EEG was obtained from 43 participants (of the 47, three participants withdrew from the study prior to completion of the post-EEG, and one participant complete post-treatment EEG but the data was not usable). The clinical symptoms were assessed via self-report symptom checklists, namely Inventory of Depressive Symptoms-Self Report (IDS-SR) [23] PTSD Checklist for DSM-5 (PCL-5) [26]. The summary changes in symptoms are reported previously [20] and reviewed again in the supplementary results.

### EEG acquisition

**Fig. 1:**
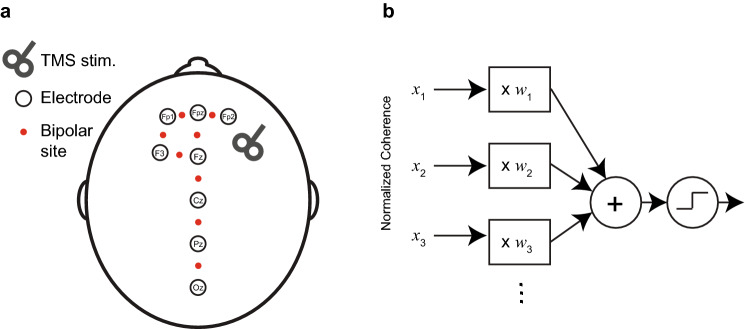
EEG electrode placement and classifier design.** a** Placement of 8 dry electrodes.iTBS was delivered over site F3, targeting the DLPFC. Red circles indicate the location of bipolar montage sites, used to avoid noise introduced by a common reference. **b** Schematic depicting a linear classifier. Classification is done by calculating a weighted sum of a set of predictor inputs (also called “features” hear marked by *x*_1_, *x*_2_, …, *x*_*n*_) and then applying a threshold to assign each of the cases to one of the two classification categories. The algorithm assigned the weights (*W*_1_, *W*_2_, …*W*_*i*_)

EEG was acquired following similar methods, as described in Zandvakili et al. [29]. In brief, resting-state eyes open and eyes-closed EEG was recorded for 10–15 min while participants were asked to sit quietly and to remain as still as possible during the recording. Participants were asked to keep eyes open for a minute, eyes closed for 10–12 min, and eyes open again for another minute. Only eyes-closed data were analyzed. Using an 8-channel electrode cap and EEG device (StarStim, Neuroelectrics, Cambridge, MA, USA), dry electrodes were placed over FP1, FP2, FPz, F3, Fz, Cz, Pz, and Oz (Fig. 1a). Electrode placement included the TMS target area, the dorsolateral prefrontal cortex (DLPFC), plus midline sites where we expected modulation in functional connectivity, informed by previous work [15]. EEG acquisition used a low-pass filter (50 Hz), a high-pass filter (0.5 Hz), sampled at 500 Hz, and was digitized at 24-bit precision.

### EEG analysis

EEG preprocessing was conducted using custom computer scripts written for MATLAB (v2019a; Mathworks, Natick, MA, USA), and using the methods described in [29]. EEG data were re-referenced through amplitude subtraction into eight nearest-neighbor bipolar electrode pairs. We used bipolar montage to avoid volume conductance and shared electrode noise (see discussion for further details on the rational). The data was then segmented into 2-s non-overlapping epochs. Epochs containing artifacts (eye movement, muscle, or movement-related) or amplifier drift were removed after manual inspection. Only data from individuals with > 120 s of usable data (sixty 2-s epochs) were used in the analysis. We also excluded EEG data when it was not recorded in all 8 electrodes, or one or more electrodes had low quality of data,this was necessary as extrapolating the signal from neighboring sites was not feasible with eight recording electrodes. 19 of 24 participants in the active treatment and 16 of 23 participants in the sham group had either pretreatment or post-treatment EEG recording that fulfilled the above criteria. Eyes closed recording was done for 10–12 min; and of the sessions that met the above criteria, there was an average of 6.8 ± 3.2 min of usable data. The power spectral density of artifact-free 2-s epochs was calculated using a Welch Power Spectral Density estimate and a Hamming window with a 50% overlap (MATLAB Signal Processing Toolbox). Power was calculated for frequency bands corresponding to delta (1–4 Hz), theta (4–8 Hz), alpha (8–13 Hz), beta (13–30 Hz), for all nearest-neighbor bipolar electrode pairs (see the list in supplementary methods).

We used coherence to quantify the statistical dependence between two EEG signals and as a tool to make inferences about functional connectivity between two recorded sites. We calculated coherence between all possible pairings of the eight bipolar recording sites (28 possible pairings for 8 recording sites). Coherence was calculated based on spectral density (estimated via Welch method and a Hamming window with a 50% overlap) using the following function:

$${C}_{x,y}\left(f\right)=\frac{{\left|{G}_{x,y}\left(f\right)\right|}^{2}}{{G}_{x,x}\left(f\right). {G}_{y,y}\left(f\right)}$$

where for each frequency, *f*, *G*_*x,y*_(*f*) is the cross-spectral density between *x* and *y*, and *G*_*x,x*_(*f*), and *G*_*y,y*_(*f*) are the auto-spectral density of *x* and *y*, respectively. |G| denotes the modulus of G. Coherence values, *Cx,y*(*f*)*,* vary between 0 and 1 with 0 indicating no statistical relationship and 1 being full coherence. Coherence values from individual bins within a frequency band were averaged to obtain the coherence for that band.

### Machine learning

The analyses were conducted using MATLAB. For the Support Vector Machine, we used the LIBLINEAR library [6] compiled in MATLAB. We also used Support Vector Machine (SVM, specifically an L1 regularized SVM implementation through LIBLINEAR [6]. In its simplest form, a linear classifier works by calculating a weighted sum of a set of predictor inputs (also called “features” or independent variables) and then applying a threshold to assign each of the cases to one of the two classification categories (Fig. 1b). Classifier algorithms, such as SVM, are algorithms that assign and find the optimal weights for the classifier. A set of weights/a trained classifier is also called a “model.” To be more specific, if the training dataset is represented as a set of values (*x*_1_*, y*_1_)*, …, *(*x*_*n*_*, y*_*n*_), where *x*_1_*−x*_*n*_ each represent a vector of features and *y*_1_*−y*_*n*_ can assume values of − 1 or + 1 to point to pre- and post-ITBS or sham vs. active session, respectively. The classifier fitted a vector of weights, *w*_*n*_ to minimize this equation:

$${\left|\left|w\right|\right|}_{1}+C{\sum }_{i = 1}^{n}{\left(\mathrm{max}\left(\mathrm{0,1}-{y}_{i}{w}^{T}{x}_{i}\right)\right)}^{2}$$

*||w||*_1_ denotes the Euclidean norm of *w* and *w*^*T*^ denotes the transpose of array *w*. The SVM algorithm uses a weighted sum of the coherence measures (28 coherence measurements in each EEG session) and minimizing the equation above by adjusting *C* to achieve the best predictive outcome.

We trained four independent sets of linear classifiers to assign EEG recordings. The classifiers were trained to separate sham vs. active participants in pre- and post-treatment groups, and also trained to separate pretreatment vs. post-treatment recordings in sham and active treatment groups. The classifiers were trained on several control contrasts to identify results related to active stimulation. This included baseline (pretreatment) sham vs. active participants, as well as pre vs. post sham treatment. The algorithm was trained on 28 features (i.e., coherence in all possible pairings of the eight recording sites). Linear SVM has one modifying parameter, C (i.e., misclassification cost, error penalty). When a small misclassification cost is used, the algorithm can find more global and generalizable trends in the data but is prone to making errors. A higher misclassification cost is associated with more specific solutions, a lower error rate, but less generalizability. The goal is a misclassification cost best balancing error and generalizability.

We thus explored misclassifications (C) values ranging from 0.1–100 (30 bins in log scale) to find the C with the best balance between error and generalizability. Classifiers were trained on a training data set comprising all EEG recordings except for one of each classifying recordings (i.e., leave-two-out cross-validation). We used leave-two out to have both conditions represented on the test set of each cross-validation iteration. This process was repeated for 500 iterations, each time leaving out two random samples (one from each group) (i.e., 500-fold). The model was trained on all the data except for the left-out samples, and the performance was examined on the test set, consisting of the two samples that were left out of training. To calculate AUC, sensitivity, and specificity, we retrain the full model with all samples and with the misclassification cost (C) that was associated with the best performance. The classifier was independently trained for the four frequency bands, defined as Alpha, Beta Theta, and Delta (Alpha: 8–13 Hz, Beta 13–30 Hz, Delta 1–4 Hz, and Theta 4–8 Hz).

To ensure observed results were not related to over-fitting, we used shuffling as an additional validation step. To do this, we randomly shuffled the EEG recordings for each classifier, training the model to use the EEG data to classify and learn the (now randomly shuffled) classification group. We repeated this process 1,000 times, each time shuffling the outcome data and rerunning the model. Similar to the original analysis, the model’s ability to predict classification group was assessed with leave-two-out cross-validation (i.e., Shuffled training set used to classify the two left-out shuffled test set and repeated for 500 iterations).

As an additional, third step of validation, we used the model trained on Active vs. Sham post-treatment and applied it to all of the recorded pretreatment EEG recordings without retraining.

## Results

**Fig. 2:**
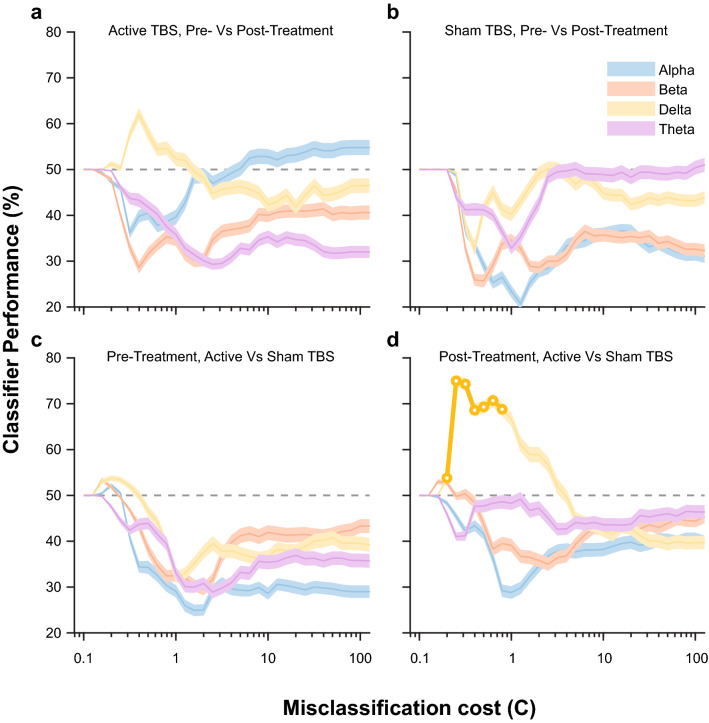
Performance of an SVM-trained classifier in assigning EEG sessions. The plots show the performance of the classifier as a function of C, the misclassification cost term. Lower C values indicate that the classifier has a broad scope and can detect global features but can undertrain, higher C values mean that the classifier relies on more global features but can overfit. Also, lower C is associated with less complex models and higher C is associated with higher model complexity. To detect an optimal C value, we assessed the performance of the classifier for a range of C values. Panel **a** is classifying pre vs. post-treatment recordings in the active group, panel d is classifying active vs. sham post-treatment sessions. Panel **c** and **b** are controls, classifying pre- vs. post-treatment recordings in the sham group, and active vs. sham group pretreatment, respectively. The points where the classifier predicted significantly are marked by a bold line

A classifier trained on EEG data was able to successfully separate patients who received active treatment vs. sham treatment (see Fig. 2d), with significant findings in the Delta band. When using coherence in the Delta band, the classifier was able to detect sham vs. active iTBS significantly (AUC = 0.89, 95% confidence interval = 0.62–0.98, sensitivity in detecting active 93%, specificity 85%, cross-validated accuracy 75.0%). We used a permutation method to assess the significance of this performance. The likelihood that this performance was due to chance was 0.2% (the equivalent of *p* = 0.002). Classifiers trained on other frequency bands did not reach significance. There was a similar trend in classifier separating pre- vs. post-treatment in the active group (i.e., to predict response to active iTBS) where the model was able to predict post-treatment EEG with a cross-validated accuracy of 62.1%, but the performance did not reach significance in permutation test (*p* = 0.088). As expected, the classifier trained on pretreatment data (i.e., active vs. sham group before treatment) and also the classifier trained on pre- and post-treatment sham group did not find any changes in coherence.

**Fig. 3:**
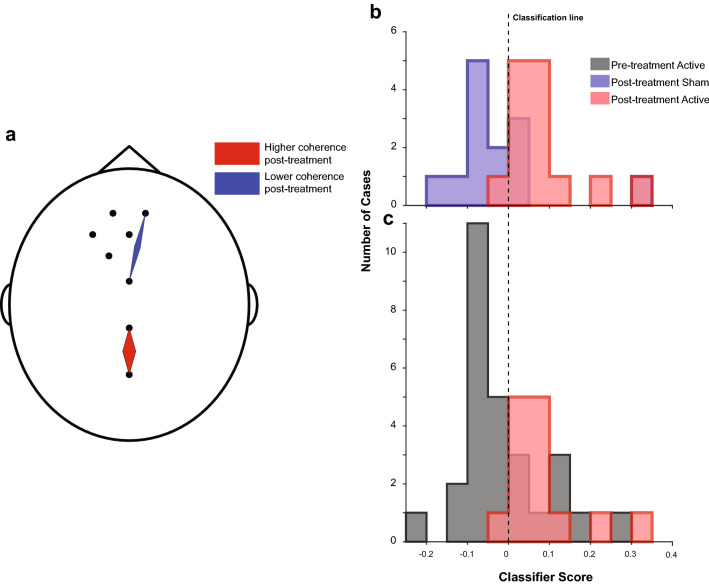
EEG functional connections and their contributions to classifier performance. The figure depicts the weights of functional connections in the classifier. The thickness of each diamond represents the weight, and red and blue represent positive and negative weights, respectively (red means that functional connectivity increased, blue means it decreased). Panel b shows the classification score—the outcome of the classifier before thresholding) and decision boundary. In our case, a positive score was associated with active and negative with sham. Panel c shows how the classifier assigned pretreatment recordings. Note that 19 out of 28 cases of pretreatment were assigned to the “sham” group

We interrogated the classifier, identifying the functional connections that were used by the model to classify EEG recordings. We only limited this to the Delta band EEG in the Active vs. Sham post-TBS group as this was the only model with statistically significant performance. As stated above, we used a regularized implementation of the SVM classifier, limiting the complexity of the model and the number of connections used in the prediction. When checking the model, we found that the model used two sets of functional connections. First, there was an increase in midline functional coherence, between central and occipital regions after iTBS. Second, iTBS was also associated with a reduction in coherence between the right frontal and central electrodes (Fig. 3a).

To demonstrate the classifier’s function, we have calculated the classifier score for all patients (Fig. 3b). The classifier assigns a score to each EEG recording, those with a positive score are classified as “treated with active iTBS,” and those with negative scores are treated with sham. Note that only 5 out of 26 recordings are misclassified (4 patients receiving sham treatment were classified as active and 1 active recipient classified as sham). We then used the same classifier (trained on post-treatment active vs. sham data) to assign pretreatment data in both active and sham groups. The classifier had not been trained on this data, and we expected that all of them would be assigned to the “sham” group as none of these EEGs are from patients treated with iTBS. Indeed, the classifier assigned 19 out of 28 of these cases (67.9% performance) to the “sham”/untreated group. The score is plotted in Fig. 3c and provided further validation of observed results.

**Fig. 4:**
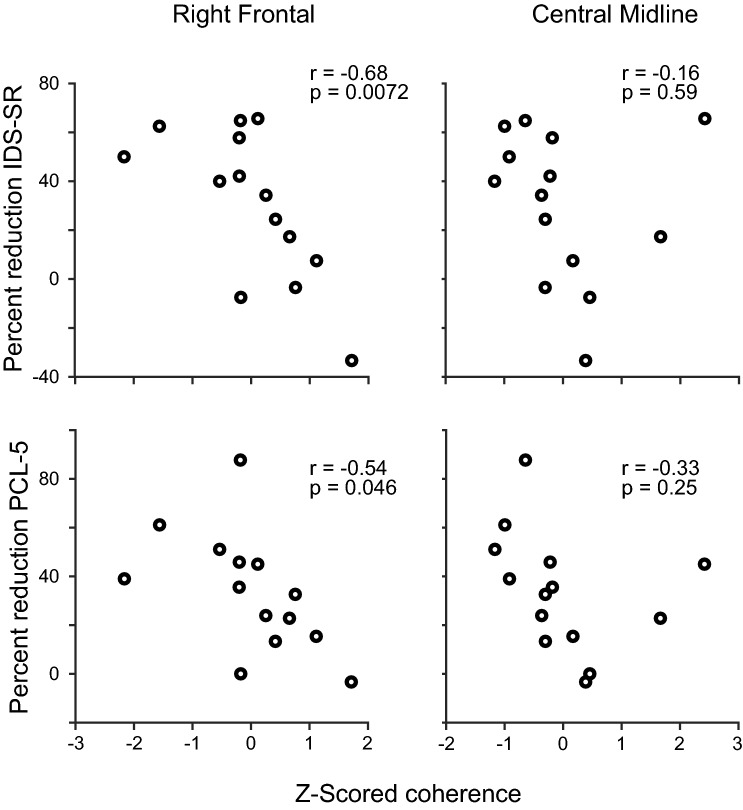
Changes in symptoms vs. post-treatment EEG coherence. We have plotted changes in symptoms, as quantified by the percent change in IDS-SR and PCL-5 vs. *Z*-scored post-treatment coherence in the two connections that had a non-zero weight in Fig. 3. Note that a higher change in IDS-SR depicts more improvement in depressive symptoms and a positive change in PCL-5 score representants improvement in PTSD symptoms. In both cases, a negative number depicts the worsening of symptoms

Finally, we assessed whether iTBS emerged changes in EEG connectivity was associated with changes in symptom severity. To this end, we assessed if post-treatment coherence in the two sets of connections that had a non-zero weight in the classifier were corrected with a change in clinical symptoms (as quantified by changes IDS-SR and PCL-5 scores, tracking depression and PTSD symptom severity, respectively). We found that a lower post-treatment right frontal connectivity was associated with higher clinical symptom improvement (for IDS-SR, *r* = − 0.68, *p* = 0.0072, for PCL-5, *r* = − 0.54, *p* = 0.046) but the same trend was not present for midline central connections (for IDS-SR, *r* = − 0.16, *p* = 0.59, for PCL-5, *r* = − 0.33, *p* = 0.25, Fig. 4).

## Discussion

This report describes a novel application of machine learning to identify EEG changes in functional connectivity, with data derived from the first sham-controlled study of iTBS for PTSD. We showed that an SVM classifier, trained on functional connectivity extracted from a sparse EEG array (i.e., 8 lead, dry electrode system), successfully identified patients who had received a course of TBS for symptoms of PTSD. The most significant contributors to the classifier involved coherence in the Delta band, at or near the site of stimulation, as well as coherence between that region and midline central-occipital sites. We also observed a statistical trend, also in the delta band, towards identifying patients most likely to respond to active iTBS. Our findings raise the possibility that EEG and machine learning could be combined inexpensively in the treatment clinic setting for ongoing therapeutic monitoring. Also, this approach can provide a window into mechanisms of action of TMS, which could inform the development of individualized treatment methods.

Our findings related to the Delta band raise important questions. To date, the majority of EEG studies of TMS have primarily focused on major depressive disorder with limited success (e.g., [27,28]. However, there is some indication EEG signals can be used to develop new TMS approaches (e.g., [11] and predict response to devices synchronized to the EEG signal (e.g., [22]. The comparable literature in EEG and brain stimulation for PTSD is much more limited. EEG studies to date have primarily focused on comparing patients with healthy controls (reviewed in [2], and integration of EEG into TMS studies is in its earliest stages [18]. Thus, prospective replication of our results will be required before they can be implemented more broadly.

However, our findings are consistent with the consensus that neural mechanisms underlying TMS involve changes in plasticity in cortical networks [8,19]. Our finding confirms that TBS treatment is associated with changes in functional connectivity that employs neural plasticity and induces changes measurable by a sparse EEG array. This is also in line with our previous finding that used the same approach in patients who received TMS treatment for comorbid PTSD and depression [29]. In that study, we found the utility of classifiers used across frequency domains, yet with the notable limitation that that prior study was not sham-controlled, making it difficult to determine whether observed findings were related to verum or non-specific (e.g., placebo) effects.

We also reported a negative correlation between theta band coherence among electrodes in the right frontal lobe and clinical improvement; patients who had lower post-treatment coherence in the right frontal were those who had a better clinical outcome. Though significant, we interpret this finding with caution. Clinical improvement is, by nature, subjective, and thus a noisy measure. As a result of this, a significantly higher sample size is needed to make an accurate statement about treatment outcome and its association with iTBS emergent EEG changes. This is in contrast to verum vs. sham dichotomy, where there is a binary and clear grouping, and thus a machine-learning algorithm can perform well with our level of sample size.

The study is unique due to the methods used. We used a powerful machine-learning algorithm (i.e., support vector machine), paired with L1 regularization, which limits the model complexity and the number of features (here, functional connections) used to distinguish the sham from the active group. We also took multiple steps to ensure that the data presented here is not due to over-fitting (meaning that the classifier just learned the pattern in the particular dataset and was not generalizable. We assessed the model using cross-validation and confirmed results using a permutation analysis (which assesses the ability to learn meaningless noise) and also did an out of sample validation (i.e., assessed the ability to classify pretreatment data that it had not been trained on). Machine learning tools are powerful, yet using them requires extra layers of vigor and validation for the results to be generalizable. We would like to propose our multi-level validation approach as a blueprint for the use of such methodology.

While the strengths of this study include its sham-controlled design and multiple levels of validation, there are several important limitations. Of note, like our earlier study, we used EEG coherence, calculated on a nearest-neighbor bipolar montage. This would mitigate many issues that affect coherence estimates of functional connectivity, such as common-noise from shared reference and issues related to volume conductance. This approach, however, can miss dipoles with certain locations and tangential orientations [9,30]. The study also used a modest sample size, although it remains the largest controlled study of its kind. It was also limited by the electrode montage, namely a dry, 8-electrode system. Such an approach is useful and practical, but having a richer electrode montage would help us make a better determination on the mechanism and specific networks affected by iTBS. Like many machine-learning studies, our results could have been affected by over-fitting, i.e., the results presented here might closely correspond to our dataset and are not generalizable. This is particularly an issue as the data was recorded in a single study site. We, however, took multiple steps to mitigate this issue (use of cross-validation, permutation analysis, and out of sample validation). The results, however, require validation in a larger, multi-site study. Because only one intervention (iTBS) was provided, we are not able to infer whether this biomarker is unique to stimulation (as opposed to a marker of non-specific response). As results were related to active stimulation and not sham, one can be reasonably confident our results can thus be attributable to a verum process that can be evaluated in future studies.

In conclusion, we applied EEG and machine learning to iTBS for PTSD in a proof-of-concept study, and obtained results indicating these methods represent viable tools to study cortical networks and potentially guide treatment. The algorithms chosen can elucidate neural mechanisms and allow us to constrain the inherent complexity of the data select electrophysiological features of relevance to therapy-induced changes. When expanded, this approach holds promise in designing treatment regimens, devices, and measurements to make screening and personalizing treatment possible in the office setting.

## Electronic supplementary material

Below is the link to the electronic supplementary material.

Supplementary file1 (DOCX 14 kb)
