# Supplementary material for: Changes in functional connectivity after theta-burst transcranial magnetic stimulation for post-traumatic stress disorder: a machine-learning study
Source: Eur Arch Psychiatry Clin Neurosci. 2020 Jul 27;271(1):29–37. doi: 10.1007/s00406-020-01172-5 (PMC7867551; doi:10.1007/s00406-020-01172-5)
Supplement: Supplementary file 1 — Supplementary file1 (DOCX 14 kb) [file 406_2020_1172_MOESM1_ESM.docx]

**Supplementary Table 1**, Participant Demographics and Clinical Outcomes

|  | **All** | **Active** | **Sham** |
| --- | --- | --- | --- |
| **n** | 47 | 24 | 23 |
| **Age, mean (*SD)*** | 51.47 (12.4) | 49.0 (12.6) | 54 (11.5) |
| **Females, n (%)** | 8 (17.0) | 5 (20.8) | 3 (13.0) |
| **Race** |  |  |  |
| White, n (%) | 40 (85.1) | 21 (87.5) | 19 (82.6) |
| Black, n (%) | 1 (2.1) | 0 (0.0) | 1 (4.3) |
| American Indian or Alaskan Native, n (%) | 1 (2.1) | 1 (4.2) | 0 (0.0) |
| Multi-Racial, n (%) | 3 (6.4) | 2 (8.3) | 1 (4.3) |
| No Response, n (%) | 2 (4.3) | 0 (0.0) | 2 (8.7) |
| **Ethnicity** |  |  |  |
| Hispanic, n (%) | 2 (4.3) | 0 (0.0) | 2 (8.7) |
| Not of Hispanic origin, n (%) | 43 (91.4) | 22 (91.7) | 21 (91.3) |
| No Response, n (%) | 2 (4.3) | 2 (8.3) | 0 (0.0) |
| **Clinical Outcomes** |  |  |  |
| PCL-5^a^ Total Score Pre^c^, mean (*SD*) | 49.7 (10.5) | 49.5 (9.5) | 50 (11.6) |
| PCL-5 Total Score Post^c^, mean (*SD*) | 37.8 (15.3) | 35.4 (14.5) | 40.3 (16.3) |
| IDS-SR^b^ Total Score Pre, mean (*SD*) | 41.2 (12.0) | 42.6 (12.2) | 39.7 (11.9) |
| IDS-SR Total Score Post, mean (*SD*) | 32.8 (14.7) | 31.1 (15.5) | 34.5 (14.3) |

Participant demographics and clinical outcomes for participants included in EEG analysis (n=47). For complete clinical outcomes for all participants, see original study (Philip et al., 2019).

^a^PTSD Checklist for DSM-5.

^b^Inventory of Depressive Symptomatology, Self-Report.

^c^Pre scores were obtained at baseline prior to beginning doube-blind iTBS treatment. Post scores obtained at completion of double-blind course of iTBS treatment.

***Electrode montage***
We re-referenced our EEG data through amplitude subtraction into eight nearest-neighbor bipolar electrode pairs. The eight sites were: Fp1-Fpz, Fpz-Fp2, Fp1-F3, Fpz-Fz, F3-Fz, Fz-Cz, Cz-Pz, Pz-Oz.
